# Biomolecule and Ion Releasing Mesoporous Nanoparticles: Nonconvergent Osteogenic and Osteo-immunogenic Performance

**DOI:** 10.1021/acsami.4c17540

**Published:** 2024-11-22

**Authors:** Azin Khodaei, Qaisar Nawaz, Zhengqing Zhu, Saber Amin Yavari, Harrie Weinans, Aldo R. Boccaccini

**Affiliations:** † Department of Materials Science and Engineering, Institute of Biomaterials, University of Erlangen-Nuremberg, 91058 Erlangen, Germany; ‡ Department of Orthopedics, University Medical Center Utrecht, 3508GA Utrecht, The Netherlands; § Regenerative Medicine Centre Utrecht, Utrecht University, 3508GA Utrecht, The Netherlands

**Keywords:** Inflammation, Immunomodulatory, Bone regeneration, Tissue engineering, Flavonoid

## Abstract

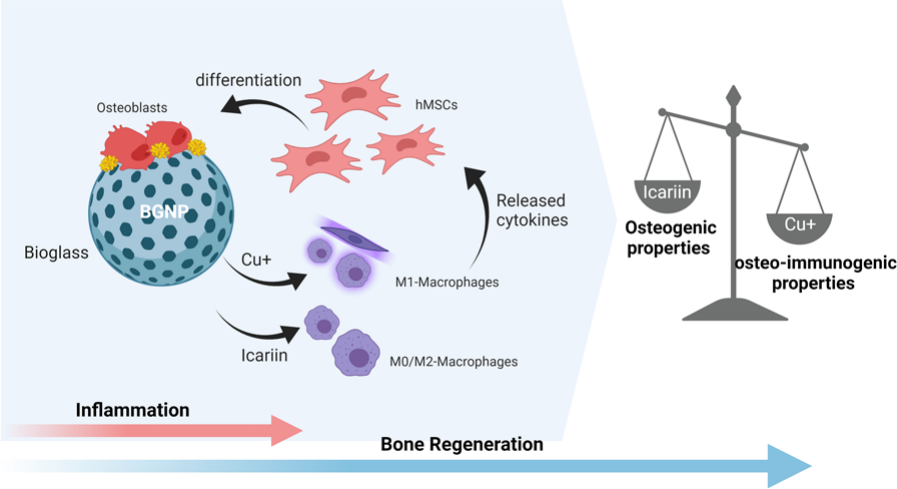

Immune-involved cell communications have recently been introduced as key role players in the fate of mesenchymal stem cells in making bone tissue. In this study, a drug delivery system for bone (re)generation based on copper-doped mesoporous bioactive glass nanoparticles (BGNPs) was developed to codeliver copper as a biologically active ion and icariin as an anti-inflammatory agent. This design was based on temporal inflammation fluctuations from proinflammatory to anti-inflammatory during bone generation. Three *in vitro* models were performed with human mesenchymal stem cells (hMSCs) to verify the osteo-immunomodulatory effects of released copper ions and icariin: nonstimulated, co-conditioned with macrophage medium and co-cultured with macrophages. Both icariin and copper showed increased levels of alkaline phosphatase activation, indicating a direct osteogenic effect. Copper-doped BGNPs showed the highest increase of osteo-immunogenic properties in a mineralization assay and also induced short-term inflammation. However, the mineralization dropped in copper doped BGNPs after loading with icariin due to copper-icariin chelate formation and inhibition of the early inflammatory phase in the immune-stimulated *in vitro* models. In the absence of copper, the direct osteogenic properties of icariin overtook its osteo-immunogenic inhibition and increased calcification. Overall, BGNPs doped with 5 mol % copper and no icariin showed the highest bone-forming capacity.

## 1 Introduction

Bioactive glass nanoparticles (BGNPs) have gained a lot of attention in orthopedic and orthodontal applications.^1,2^ These amorphous biomaterials can interact with cells and the surrounding tissue by releasing bioactive ions previously doped in the silicate structure such as calcium, zinc, magnesium, strontium, or copper. When body fluids wet the surface of BGNPs, hydroxycarbonate apatite (HCA) forms on the particles’ surface.^3^ A variety of biomaterials have been developed incorporating BGNPs as the bioactive and reinforcing agent. Among all the elements considered to enhance the biological activity of BGNPs, copper (Cu) as a well-known osteogenic, angiogenic, and antibacterial element is favorable for bone regeneration and repair.^4^ Regarding the very early interaction of Cu with the immune system, it is believed that Cu ions induce inflammatory responses.^5^ Moreover, it is well-known that the crosstalk between immune cells, bone cells and their precursors, the mesenchymal stromal cells (MSCs), plays a key role in bone remodeling and regeneration.^6−9^ During the first phase of bone healing, the “inflammation phase”, innate and adaptive cells including macrophages migrate to the bone defect site and develop an inflammatory environment. This phenomenon is strengthened once a biomaterial is implanted. The secreted inflammatory cytokines such as TNF-α, IL-6, IL-8, and IL-17 that result from the immune response recruit and differentiate MSCs into osteoblasts.^10^ In the second phase, known as the “repair phase’, cytokines like IL-10 and IL-4 reverse this and create an anti-inflammatory environment that inhibits osteoclastogenesis and further stimulates osteoblasts leading to bone tissue formation and mineralization. Mimicking this natural timeline provides an opportunity to take advantage of evolutionary perfection in fracture healing and bone regeneration.

A previous study on Cu ions showed concentration-dependent immunomodulatory properties.^11^ Concentrations lower than 100 μM could turn the macrophages into the M2 phenotype (anti-inflammatory), while higher concentrations stimulated the M1 (pro-inflammatory) phenotype.^11^ In another in vitro study, copper-incorporated silica nanoparticles also showed immunomodulatory effects and promoted osteogenesis.^12^ However, although the in vitro model included the addition of immune conditioned medium to MSCs, the interaction between immune cells and MSCs was left out.^12^ So far, the main focus in immunomodulatory strategies has been on anti-inflammatory drugs and their immune reactions for restraining ROS levels increase and osteoclastogenesis.^13^ Icariin (Ic), as the bioactive flavonoid compound extracted from the Chinese herb Epimedium, is an ancient drug known for modulating osteogenesis, angiogenesis and anti-inflammatory properties.^10,14^ However, the osteogenic properties of different concentrations of this drug have been studied through MSCs-based in vitro models, which in some cases are not translatable to the in vivo situation.^15^ Multiple studies on surface-modified and loaded biomaterials with icariin have shown increased osteogenesis.^16^ Developing a biomaterial consisting of both inflammatory and anti-inflammatory agents with controlled release based on an immune activity timeline in natural bone healing can boost osteogenesis to another level.

In this study, mesoporous Cu-doped BGNPs (containing 3 and 5% copper) were used as drug carrier to codeliver icariin. Three in vitro models with and without the involvement of macrophages were used to investigate the effect of the combination of the inflammatory and anti-inflammatory properties of icariin and copper on the osteogenic and immunomodulatory properties of BGNPs with codelivery of Cu and/or icariin.

## 2 Materials and Methods

### 2.1 Synthesis of BGNPs

The microemulsion modified sol–gel method was used to synthesize mesoporous BGNPs as previously reported.^17^ For this purpose, cetyltrimethylammonium bromide (CTAB, ≥97%) was dissolved in Milli Q water (<2000 μS/cm) with 0.02 mg/mL concentration. The solution was kept stirring for 30 min at 37 °C to become homogenized. Then, the heater was turned off and 16 mL of ethyl acetate (EA, ≥99.8%, Sigma-Aldrich) was added to 52 mL of the solution while left stirring for 30 min. CTAB as a surfactant and EA as the organic solvent produced a microemulsion in this step to be used as a soft template to induce mesoporosity formation in the final product. 1.4 mL of 28% ammonia was added afterward to catalyze the reaction and adjust the basic pH (10.20–10.40). 6.65 mL of tetraethyl orthosilicate (TEOS, 98%, Sigma-Aldrich) was added to the reactor in this step. To add Ca to the structure, 2.5 g of calcium nitrate tetrahydrate (≥99.4%, VWR) was added to the reaction and kept stirring for 4 h. At the end, the precipitate was washed three times with ethanol and water, dried at 60 °C and was calcinated at 650 °C for 3 h. The final product was ground and sieved using 100 mesh (150 μm).

To dope nanoparticles with Cu and prevent copper oxide formation, a complex of Cu/l-ascorbic acid was used as a precursor. To prepare this complex, 50 mL of 0.2 M CuCl_2_.2H_2_O (≥99.99%, Sigma-Aldrich) was dissolved in a round-bottom flask in an 80 °C oil bath. After 1 h of stirring, 50 mL of 0.4 M l-ascorbic acid (≥99%, Sigma-Aldrich) was added dropwise. After 24 h of incubation at 80 °C, the brownish precipitation was collected and centrifuged and the supernatant was stored at 4 °C for further use. To add 3 and 5 mol % of Cu to the BGNPs structure, 3 and 5 mL of Cu/l-ascorbic acid was added after calcium nitrate addition. Commercial nanohydroxyapatite powder (Sigma-Aldrich) was also used in in vitro studies as a control.

### 2.2 Preparation of Icariin-Loaded BGNPs

To load icariin (>96.0%, TCI chemicals) into the pores of the BGNPs, a dimethyl sulfoxide (DMSO) based solution containing 0.15% icariin was used. The nanoparticles were dispersed at a concentration of 0.03 g/mL and the suspension was kept stirring for 1 h. After that, the particles were collected and washed three times with DMSO and water using a 4000 rpm centrifuge for 5 min. In the end, the loaded nanoparticles were lyophilized for characterization. Furthermore, icariin showed chelate formation ability with Cu ions which ended in green precipitation formation. To investigate the properties of the complex, 0.03 g/mL icariin in DMSO was incubated with 5 M CuSO_4_·5H_2_O with a ratio of 1:4. The precipitation was washed and lyophilized, accordingly.

### 2.3 Characterization of BGNPs

Transmission electron microscopy (TEM, FEI Tecnai 12) was used to visualize the synthesized nanoparticles. The samples were made ready by dispersing nanoparticles in ethanol and locating the carbon-coated (2 nm) copper grid on top of the droplet. To confirm the amorphous structure of the particles and lack of impurity, the powder X-ray diffraction (XRD, Rigaku, MiniFlex 600, Japan) method was applied. Raman spectroscopy was conducted by using a spectrometer (LabRAM 800, HORIBA, Jobin Yvon) equipped with a red laser (He-Ne) with an excitation wavelength of 633 nm. A microscope objective and grant of 50× and 1800 grooves/mm were used to record the spectra, respectively. The actual concentration of Cu doped in the samples was determined using Inductively Coupled Plasma- Optical Emission Spectrometry (ICP-OES). The same method was also used to determine the released concentration of Cu from the samples after 1, 3, and 7 days of incubation.

After loading BGNPs with icariin, attenuated total reflectance Fourier transform infrared spectroscopy (ATR-FTIR, IRAffinity-1S, Shimadzu) was used to confirm the drug loading and the chemical bonds in drug-loaded samples. UV–vis spectroscopy (Clariostar plate reader, BMG Labtech) was used to study the absorption spectrum of icariin in the presence and absence of copper ions. The surface charge of the nanoparticles was also quantified using a Zetasizer Nano-Z (Malvern, UK) instrument.

### 2.4 Metabolism and Cell Viability Study

Table 1 represents the in vitro experimental groups including three groups of BGNPs with nominally 0, 3, and 5 mol % Cu, before and after icariin loading. Nanohydroxyapatite (nHA) before and after loading with icariin was considered in these studies, as well as Ic-Cu chelates.

**Table 1:** In Vitro Experimental Groups (Coding and Compositions)

Drug loading status	Sample code	Composition
Without Icariin	BG	70SiO_2_ −30CaO (mol %)
	BG3%	70SiO_2_ −27CaO- 3CuO (mol %)
	BG5%	70SiO_2_ −25CaO- 5CuO (mol %)
	nHA	Ca_10_(OH)_2_(PO_4_)_6_
With Icariin	BG-Ic	70SiO_2_ −30CaO (mol %)-Icariin
	BG3%-Ic	70SiO_2_ −27CaO- 3CuO (mol %)-Icariin
	BG5%-Ic	70SiO_2_ −25CaO- 5CuO (mol %)-Icariin
	nHA-Ic	Ca_10_(OH)_2_(PO_4_)_6_-Icariin
	Ic-Cu	Icariin- Cu
	Cu	

As copper ions can cause cytotoxicity, Alamar blue assay and live–dead staining were used to verify the noncytotoxic concentration of released supernatant from different experimental groups. Three concentrations of 0, 0.1, and 0.5 mg/mL nanoparticles were studied to find the safe concentration for bone marrow-derived hMSCs and the THP-1 human leukemia monocytic cell line. As THP-1 derived macrophages showed higher sensitivity, the results for this cell type are described in the following.

For this purpose, first, THP-1 cells were cultured in a 75 mL flask with advanced RPMI 1640 medium (Invitrogen, USA) containing 10% (v/v) FBS and 1% (v/v) Pen-Strep (Invitrogen, USA). To transfer cells to well plates and differentiate them into attachable M0 phenotype macrophages, the cells were dispersed in a medium containing 160 nM phorbol 12-myristate 13-acetate (PMA, ≥99% Sigma-Aldrich). Subsequently, 80,000 THP-1 cells/well were cultured in a 48-well plate. After 24 h, the medium was exchanged with three concentrations of the experimental groups. The metabolic activity of the cells was quantified after 72 h using an Alamar blue kit (Molecular probes, ThermoScientific, US) following the manufacturer’s protocol. Cells were incubated with 0.01 mg/L of resazurin salt in medium for 4 h. The condition medium was plated, and the fluorescent intensity was quantified with excitation and emission wavelengths of 530 and 590 nm, respectively. To verify the direct correlation between metabolic activity and cell viability, cells were stained with a live–dead kit (Molecular Probes, ThermoScientific, US). A confocal laser microscope (CSLM-Leica SP8X, Germany) was used to image the signals of live and dead cells in two colors; green: 500–525 nm, and red: 528–640 nm, respectively. It is worth mentioning that all samples were exposed to UV light for 30 min in a dry state for sterilization.

### 2.5 Osteogenesis and Osteo-immunogenesis Study

**Figure 1:**
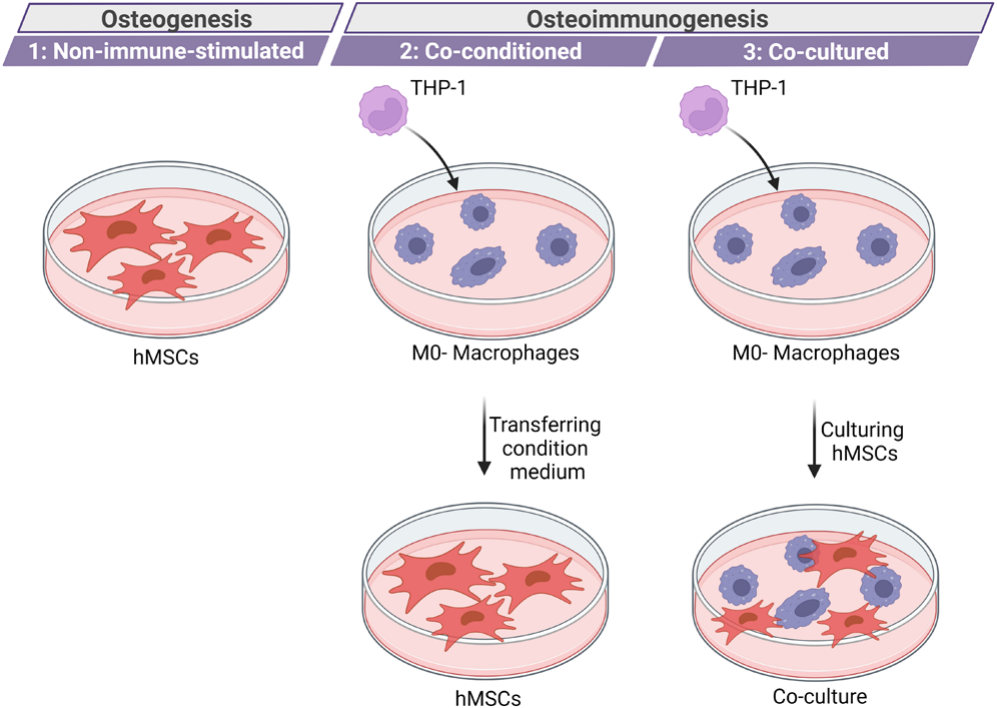
Three *in vitro* models used in this research to study osteogenesis and osteo-immunogenesis, including: (i) a non-immune-stimulated model to study osteogenesis; (ii) a co-conditioned model with transfer of condition medium from macrophage culture, and (ii) a co-cultured model combining MSCs and macrophages.

To study and compare the osteogenic and osteo-immunogenic properties of released ions and drugs from loaded and nonloaded BGNPs, the released supernatant of the particles was introduced to the cells in three in vitro models. To collect the supernatant, dried samples with 0.1 mg/mL concentration were dispersed in α-MEM (Invitrogen, USA) with 10% (v/v) FBS and 1% (v/v) Pen-Strep (Invitrogen, USA). The samples were then incubated at 37 °C with a stable CO_2_ level (5%). The supernatant at every time point was collected after centrifuging the tubes for 5 min at 1500 rpm. At each time point, the medium was refreshed after sample collection to mimic the gradient release of the agents from the biomaterial after implantation into the body. Figure 1 represents these three in vitro models.

#### 2.5.1 Non-immune-Stimulated Model

A simple 2D in vitro model including hMSCs (maximum passage 6) was used to investigate the indirect effect of experimental groups based on the released Cu^2+^ ions and icariin. To culture hMSCs, they were dispersed in complete α-MEM medium at a density of 10,000 cells/well in a 48-well plate. After 24 h of incubation, the condition medium was replaced with the supernatant collected from the samples.

#### 2.5.2 Co-conditioned (Immune Stimulated) Model

In this model, two cell types, THP-1 derived macrophages and hMSCs, were cultured in different well plates. THP-1 derived macrophages were incubated with the supernatant, and after 24 h, the condition medium was collected and introduced to hMSCs. To do so, 120,000 THP-1 cells/well were cultured and differentiated in a 24-well plate, as mentioned before. After 24 h of differentiation, RPMI medium was replaced with α-MEM. After 4 h of incubation, the medium was replaced with supernatant collected from each experimental group. The cells were left incubated for 24 h, and then the condition medium was moved to hMSCs well plates. hMSCs were cultured as previously described. The collected supernatant at different time points was introduced to independent wells of THP-1 derived macrophages for 24 h, while the hMSCs were exposed to the condition medium over time after 1, 3, and 7 days of incubation.

#### 2.5.3 Co-culture Model

The third in vitro model included the co-culture of THP-1 derived macrophages and hMSCs. At first 50,000 THP-1 cells/well were cultured and differentiated in a 48-well plate. After 24 h of differentiation, RPMI medium was replaced with α-MEM. After 4 h, the medium was suctioned out and 10,000 hMSCs cell/well were cultured on top of THP-1 derived macrophages. After overnight incubation, the experimental groups were added indirectly (released supernatants collected over time after 1, 3, and 7 days of incubation) or directly.

### 2.6 Alkaline Phosphatase (ALP) Activity

To study osteogenic differentiation, the activation of ALP was quantified after 10 days of incubation using a colorimetric assay kit (Abcam, UK). This kit is based on the property of ALP to hydrolyze the substrate para-nitrophenylphosphate (PNPP).^18^ To prepare the samples, cells were washed with PBS and then 200 μL of lysis buffer (0.2% Triton-x 100 in PBS) was added to each well and left incubated at room temperature for 30 min. 100 μL of this solution was incubated with PNPP buffer for 10 min, and its optical density was quantified at 405 nm and corrected to 655 nm optical density. A calibration curve of pure ALP was used at the end to determine the concentration of activated ALP in each well. As the amount of ALP in each well is a function of cell number, the results were normalized to the DNA content in each well. For DNA quantification, the same plates were freeze–thawed three times, and then a Quant-iT PicoGreen kit (ThermoFisher Scientific, USA) was used based on the manufacturer’s protocol. For the co-cultured samples, the DNA count of the THP-1 control was subtracted from the quantified DNA count, and then ALP was normalized to that.

### 2.7 Calcification

To compare the matrix mineralization, the plates were kept incubated for 28 days. To do the assay, first, the cells were fixed with 100% ethanol for 15 min. Then the matrix was stained using 2% (w/v) Alizarin red S solution at pH 4 (Sigma-Aldrich) for 10 min. In the following, the wells were washed with PBS three times. The wells were imaged using an SZ61/SZ51 stereomicroscope (Olympus, Japan). To quantify the Ca^2+^ chelated Alizarin, 10% cetylpyridinium chloride (CPC, Sigma-Aldrich) was added and incubated for 60 min. Absorbance at 595 nm, corrected at 655 nm, was quantified and translated to concentration based on a calibration curve.

### 2.8 Immunogenicity Study

To investigate the immune response to released Cu ions and icariin, the released supernatant from 0.1 mg/mL of samples in the culture medium was introduced to THP-1 derived macrophages. As the morphology of the cells depends on the phenotype, it was studied through a confocal laser microscope (CSLM-Leica SP8X, Germany) after cytoskeleton staining. In this regard, tetramethylrhodamine B isothiocyanate (TRITC)-labeled phalloidin (Sigma-Aldrich) and DAPI (Abcam) were used to stain the actin and nuclei of the cells in red and blue, respectively. Interleukin 6 (IL-6) as an inflammatory cytokine was quantified after 24 h of incubation with released supernatant collected on days 1, 3, and 7. A human IL-6 ELISA kit (DuoSet, R&D systems) was used to quantify this cytokine based on the suppliers’ protocol.

### 2.9 RNA Extraction and qRT-PCR Analysis

Quantitative Reverse Transcription Polymerase Chain Reaction (qRT-PCR) was used to detect mRNA expression levels of Runx2 as a bone regeneration signature gene. In this regard, the co-culture model and osteogenic medium containing 50 mM ascorbic acid, 10 mM b-glycerophosphate, and 0.1 mM dexamethasone was used. The housekeeping genes were GAPDH and YWHAZ. The total RNA of the cells was extracted using Trizol (Thermo Fisher Scientific, USA), and the iScript cDNA synthesis kit (Bio-Rad, USA) was used to reverse transcribe the extracted 1 μg of RNA into 20 μL cDNA, diluted to 250 μl. The total volume of the amplification-reaction system (each well) was 15 μL, including 7.5 μL of iTaq Universal SYBR Green Supermix (Bio-Rad, USA), 2.5 μL of diluted primers in total, and 5 μL of cDNA. The conditions were 40 cycles of denaturation at 95 °C for 15 s and annealing/extension at 60 °C for 30 s, while for the YWHAZ gene, the annealing/extension was done at 65 °C. The control group was normalized by the 2^–ΔΔCt^ method and compared with other groups to determine the expression level of the target mRNA. Table 2 represents the used primers.

**Table 2:** The Primers Used for qRT-PCR Analysis

Primer	Sequences 5′-3′
Runx2	forward: ATG-CTT-CAT-TCG-CCT-CAC
	Reverse: ACT-GCT-TGC-AGC-CTT-AAA-T
GAPDH	forward: CAA-GAT-CAT-CAG-CAA-TGC-CT
	Reverse: CAG-GGA-TGA-TGT-TCT-GGA-CAG
YWHAZ	forward: ACT-TTT-GGT-ACA-TTG-TGG-CTT-CAA
	Reverse: CCG-CCA-GGA-CAA-ACC-AGT-AT

### 2.10 Statistics

All reported data were collected in triplicate, reported as mean ± standard deviation and statistically analyzed using two-way ANOVA and Bonferroni’s multiple comparison hypothesis. The asterisks in all the graphs represent the following: *P* < 0.05 (*), *P* < 0.01 (**), *P* < 0.001 (***), *P* < 0.0001 (****).

## 3 Results and Discussion

**Figure 2:**
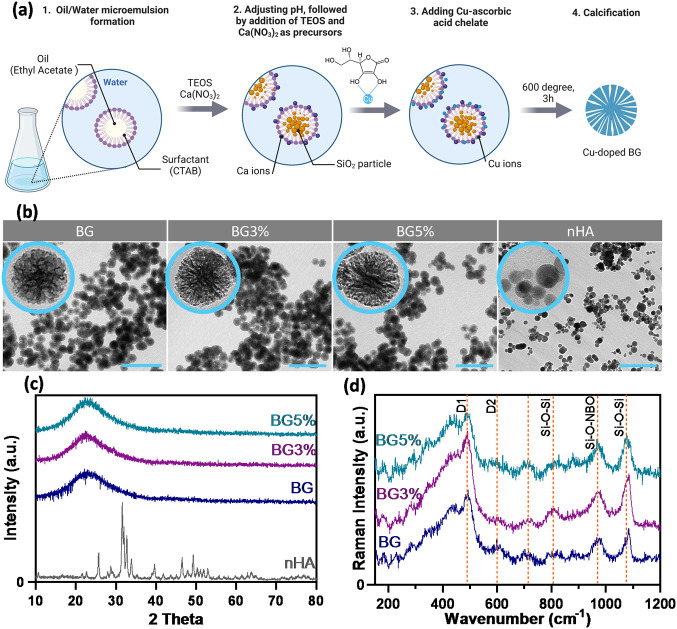
(a) Schematic diagram showing different steps of BGNPs synthesis. (b) TEM images from different experimental groups with different Cu concentrations of 0, 3 mol% and 5 mol% (groups BG, BG3%, BG5%) and a nanohydroxyapatite group (nHA). The scale bar shows 500 nm length. (c) XRD patterns of amorphous BGNPs and crystalline nHA. (d) Raman spectroscopy analysis of BG compared to copper doped samples.

The morphology and particle size of the synthesized nanoparticles were evaluated using TEM (Figure 2b). Independent of copper dopant, BGNPs and nHA showed spherical shapes with particle sizes of 97 ± 5 nm and 36 ± 14 nm, respectively. BGNPs showed radial mesoporous structures, whereas nHA particles were dense. To explain the mesoporous structure of BGNPs, it is important to recall their mechanism of formation. Ethyl acetate as the oil phase and CTAB as the surfactant formed a microemulsion, which can be considered as a soft template causing mesoporosity. The base-catalyzed sol–gel method was applied to initially form SiO_2_ nanoparticles.^19^ Calcium and copper ions were absorbed on the surface in the following steps and consequently incorporated into the structure after aging and calcination (Figure 2a). There are two main challenges involved in doping bioactive glass: oxidation of the dopant (the formation of CuO nanoparticles) and changes in the amount of the dopant compared to the stoichiometric amounts. XRD patterns (Figure 2c) were used to show that the formed BGNPs are amorphous and the Cu ions did not form crystalline CuO nanoparticles. The broad peak at around 2Θ = 23 degrees is the main characteristic of amorphous silica glasses.^20^ Due to the use of the Cu/l-ascorbic acid complex as the copper source, no sharp peak presenting the CuO phase was observed. In comparison, nHA particles showed a crystalline structure with the characteristic peaks of a hexagonal structure.^21^ Raman spectroscopy was also conducted to investigate the formation of CuO, considering the fact that the detection limit of this method (98%) is higher than that of XRD (95%).^22,23^ The Raman spectrum (Figure 2d) for BG showed main bands at 801, 956, and 1080 cm^–1^. These peaks represent Si–O–Si bending, Si–O–NBO stretching, and Si–O–Si asymmetric stretching vibrations.^24^ The broad peak at 438 cm^–1^ is characteristic of silicate synthesized by the sol–gel method. The defect lines D1 and D2 showed the symmetric breathing modes of regular 4-membered and 3-membered planar silica rings, respectively.^25^ No characteristic peak of CuO was recognized, which confirms the incorporation of copper ions in the silica structure.

**Figure 3:**
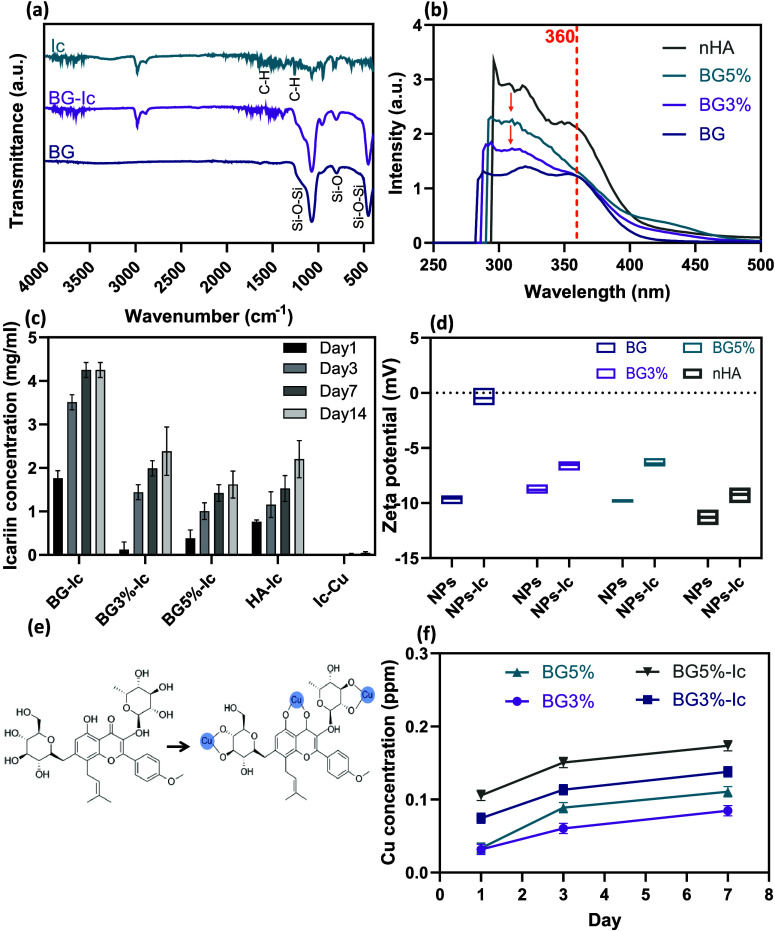
(a) FTIR spectra of BG, BG-Ic and Ic. (b) UV–vis spectra of the residue icariin in DCM after drug loading. (c) Release profile of reactive icariin from the experimental groups in PBS, determined by the folin ciocalteu calorimetric method. (d) Zeta potential of the experimental groups before and after Ic loading. (e) Mechanism of interaction of icariin with Cu^+^ ions to form Ic-Cu chelate. (f) Release profile of Cu ions from doped experimental groups determined by the ICP-OES method. All the measurements for parts (c), (d), and (f) were done in triplicate and reported as mean ± standard deviation.

After nanoparticles were loaded with icariin, the ATR-FTIR spectra of the loaded and nonloaded samples were used to verify the loading of the drug. In BGNPs, peaks at 440 cm^–1^, 800 cm^–1^, and 1070 cm^–1^ correspond to the bending vibration of Si–O–Si, symmetric stretching vibration of Si–O, and asymmetric stretching of Si–O–Si, respectively.^26,27^ Comparing it with BG-Ic, characteristic peaks of icariin were detected, proving the effective loading of the drug. These peaks were distinguishable at 1261, 1606, and 2940 cm^–1^, demonstrating the -C–H vibration in the methylene group, benzene ring, and -C–H vibration in the methoxyl group, respectively^28^ (Figure 3a). The doped concentration of Cu ions was determined using the ICP-OES method to be equal to 1.0 and 1.7 mol %, which is less than the stoichiometric ratios.

UV–vis spectroscopy was conducted on the icariin residue in DCM supernatant after drug loading to determine the loading efficiency in the different experimental groups (Figure 3b). Two characteristic peaks of icariin were recognized at 320 and 360 nm. The peak at 360 nm was considered to determine the residue concentration based on the literature.^29^ The loading efficiency of BG was calculated to be 32%, accordingly. However, the peaks shifted to lower wavelengths in the samples doped with Cu (change of color from yellow to green). This observation can be explained based on the icariin’s ability to form chelate with transition metals. Icariin is a polyphenolic flavonoid containing phenolic hydroxyl and carbonyl groups. These functional groups can coordinate metallic ions and form a chelate.^30^Figure 3e shows the mechanism explaining how the icariin molecule makes a complex with Cu ions. Because of chelate formation, it was not possible to accurately estimate the loading efficiency of icariin in copper-doped samples. The loading efficiency of nHA was determined to be three times less than that of BG and confirmed the promotive effect of mesoporosity on BGNPs’ drug loading efficiency.

Icariin release from the loaded nanoparticles and Ic-Cu chelates was determined in PBS (Figure 3c). It is believed that the capacity of flavonoids to scavenge reactive oxygen species (ROS) through hydrogen bonding is the main key to their anti-inflammatory performance. As this property comes from the hydroxyl groups in the structure, it is expected that metal chelation reduces the reactive sites of the drug.^31^ To determine the concentration of reactive icariin, the folin ciocalteu method was used. This method is based on the oxidation of phenolic compounds in an alkaline solution in the presence of molybdotungstophosphate hetero polyanion.^32^ In this procedure, Mo(VI) reduces to Mo(V) and oxidizes the phenolic hydroxyl groups.^33^ Therefore, it quantifies the reactive molecules of icariin. Looking at the release behavior, BG showed the highest release concentration over time. Having the same structure and mesoporosity, the reduced concentration of reactive icariin released from BG3% and BG5% can contribute to the chelate formation and particle coordination by icariin. This fact was confirmed by the very low concentration of released active icariin from the Ic-Cu sample. In the case of nHA, a lower concentration of released icariin was expected due to the nonporous structure and lower loading efficiency of these nanoparticles. The effect of icariin loading on the surface charge of the nanoparticles was also studied using a zetasizer (Figure 3d). The surface charge of all experimental groups was negative and moved closer to zero after loading icariin.

The concentration of released Cu was also determined using the ICP method. As expected, an increased dopant concentration led to an elevated released ion content. The released concentrations from the icariin-loaded samples (BG5%-Ic and BG3%-Ic) were higher compared to that of nonloaded samples. This result can be explained by the chelate formation ability of icariin, which likely extracts Cu ions from the particle structure. In the ICP-OES method, the aqueous solution passes through a plasma, which degrades the chelates and excites the element. Therefore, the concentration determined by ICP-OES includes the ions in the chelates.

**Figure 4:**
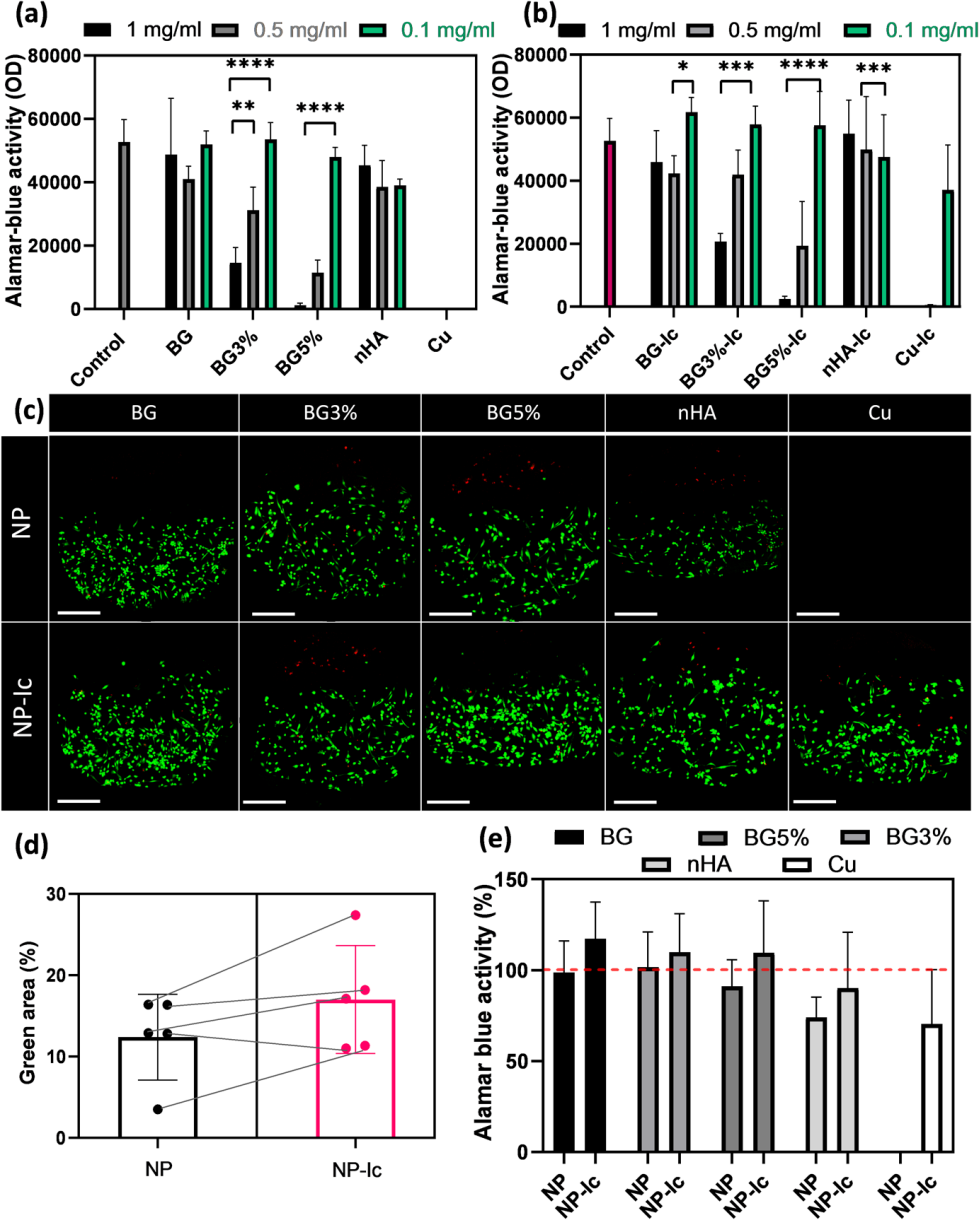
(a) Alamar blue activity of THP-1 derived macrophages incubated with the released supernatant of nonloaded nanoparticles and (b) icariin-loaded nanoparticles. The control here was THP-1 derived macrophages and the Cu concentration was 1 M (the same concentration that was used for chelate preparation). (c) Confocal images of THP-1 derived macrophages incubated with released supernatant of 0.1 mg/mL experimental groups after live (green)–dead (red) staining. (d) Green area percentage in the live–dead images which correlates with the number of alive cells. (e) The percentage of Alamar blue activity calculated based on the control group, compared between nonloaded and icariin loaded samples. All the measurements for parts (a), (b), (d), and (e) were done in triplicate and reported as mean ± standard deviation. *P* < 0.05 (*), *P* < 0.01 (**), *P* < 0.001 (***), *P* < 0.0001 (****).

Cu ions are cytotoxic to cells at specific concentrations. Alamar blue assay was applied to probe the metabolic activity of cells in the presence of released ions and drugs from different experimental groups. As THP-1 derived macrophages showed higher sensitivity to Cu ions compared to hMSCs (data not reported), they were used for the cytotoxicity study. Between three concentrations of 1, 0.5, and 0.1 mg/mL experimental groups, only the lowest concentrations of BG3% and BG5% did not affect the metabolic activity of the cells negatively. However, BG and nHA were not cytotoxic at any concentration (Figure 4a). The Cu solution decreased the metabolic activity to zero, as expected (Figure 4a). Meanwhile, the metabolic activity of the cells remained high in the presence of 0.1 mg/mL of Ic-Cu chelate (Figure 4b), which confirms the icariin release profile and the stability of the chelate.

A live–dead assay was performed using THP-1 derived macrophages incubated with 0.1 mg/mL from different experimental groups. Confocal images showed no significant difference caused by copper ions in icariin-loaded and nonloaded groups (Figure 4c). The green area (live cells) in the images was quantified by using ImageJ software. Comparing the results for loaded and nonloaded nanoparticles, it is observed that icariin was able to increase the viability of the cells (Figure 4d). Increased metabolic activity of the cells in the presence of icariin is also shown in the Alamar blue results (Figure 4e).

**Figure 5:**
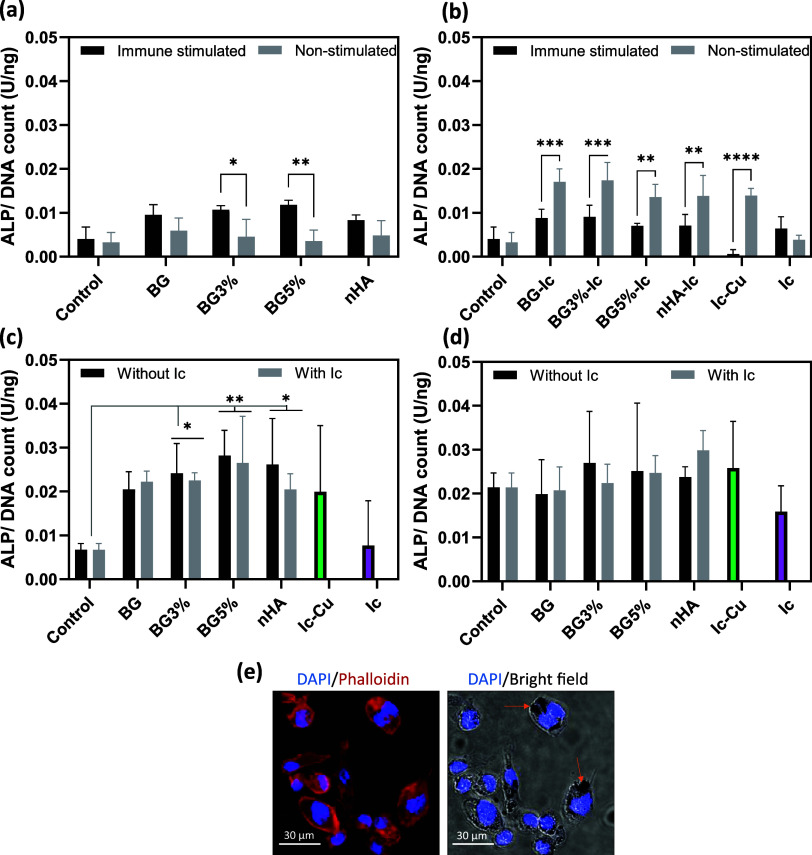
ALP normalized to picogreen (DNA count) of hMSCs incubated with (a) nonloaded and (b) icariin-loaded nanoparticles in non-immune-stimulated and co-conditioned (immune-stimulated) *in vitro* models. (c) ALP activity of hMSCs incubated indirectly (with released supernatant) and directly (d) in the co-culture model. (e) Confocal/optical images of THP-1 derived macrophages incubated directly with BG5% nanoparticles. All the measurements for parts (a) to (d) were done in triplicate and reported as mean ± standard deviation. *P* < 0.05 (*), *P* < 0.01 (**), *P* < 0.001 (***), *P* < 0.0001 (****).

In the following, the ALP activity of hMSCs was quantified in three sets of experiments with nonimmune stimulated, co-conditioned, and co-cultured in vitro models (Figure 1 shows the three models considered). The indirect (with released supernatant) study of the nonloaded nanoparticles showed an increase in ALP activity in the co-conditioned (immune-stimulated) model compared to the nonstimulated model. This elevation increased with increasing Cu dopant concentration in BGNPs (Figure 5a). However, ALP levels decreased after immune stimulation in the groups indirectly incubated with icariin-loaded nanoparticles and Ic-Cu. Icariin release in the nonstimulated condition was shown to increase ALP activity up to two times, which confirms the intrinsic osteogenic properties of this agent (Figure 5a, b).

The indirect incubation was also applied to the co-culture model of hMSCs and THP-1 (Figure 5c). In this set of experiments, no significant difference between loaded and nonloaded nanoparticles was recognized. Although all experimental groups showed an increase in ALP activity compared to the control (hMSCs incubated in growth medium), this increase was more significant in the BG5% and BG5%-Ic groups. To determine the effect of particle size, morphology, and charge, all experimental groups were also added to the co-culture model directly (Figure 5d). The ALP activity in this model was in the range of the indirect study in the co-culture model. The icariin-loaded BGNPs containing Cu ions, nHA, and Ic-Cu showed the highest ALP activity in comparison to the control. However, the average ALP level caused by bare BGNPs was also higher than that of the control. No significant difference could be validated due to the high variations. Cellular uptake of the NPs by THP-1 derived macrophages was studied using confocal and optical microscopy (Figure 5e). BG5% was selected as a model experimental group. The particles were accumulated in the cytoplasm around the nuclei, which represents cellular uptake. This behavior might affect the immune response and hMSCs differentiation consequently.

**Figure 6:**
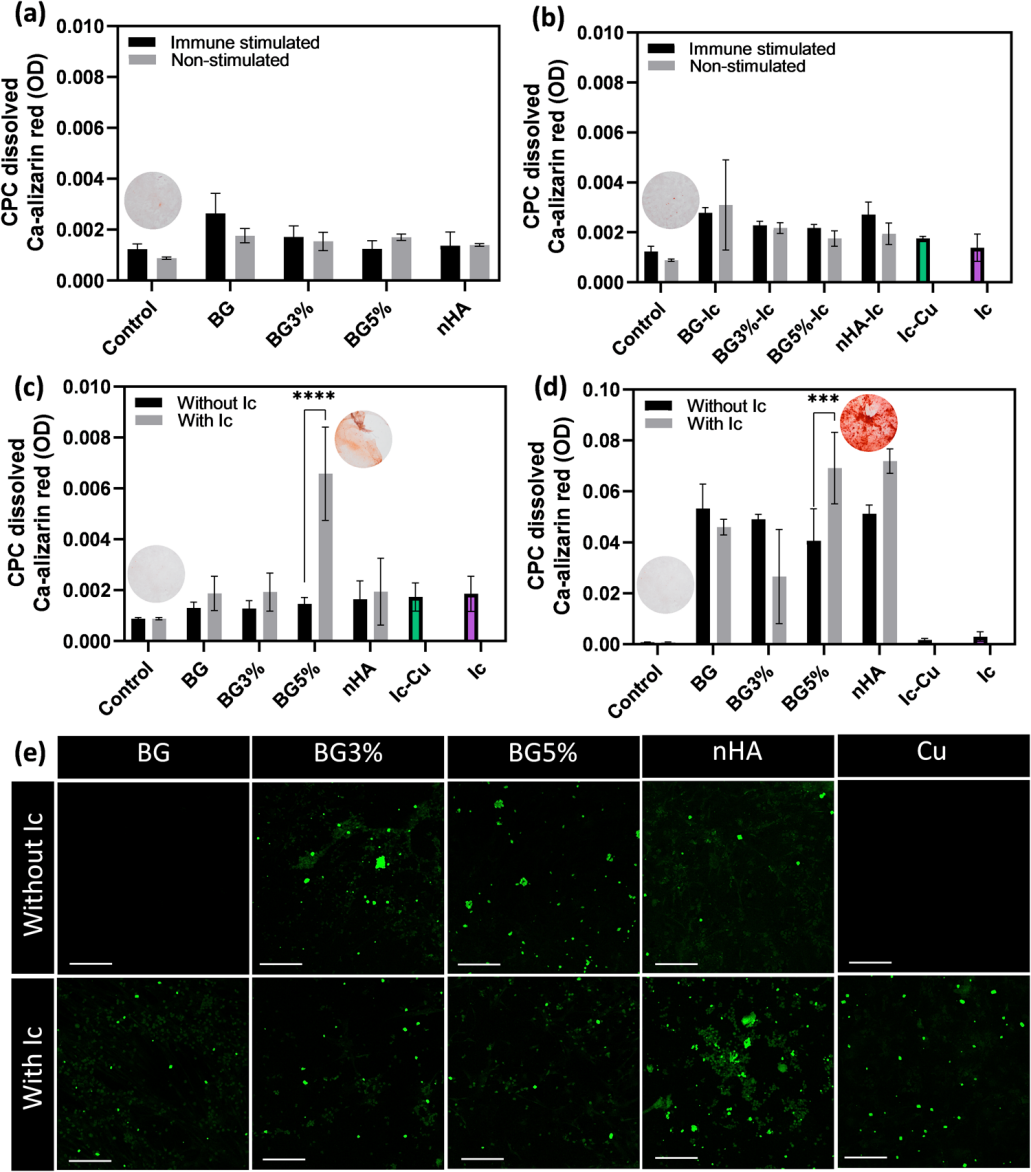
CPC dissolved Ca-Alizarin red chelate light absorbance (655 nm) for indirect test on the samples (a) without icariin and (b) with icariin in the co-conditioned (immune stimulated) and nonstimulated models. The same parameter for (c) indirect and (d) direct tests of the experimental groups in the co-culture model. (e) Osteoimages of the co-culture model directly incubated by the experimental groups: calcified hydroxyapatite stained in green (scale bar is equal to 200 μm). All measurements for parts (a) to (d) were done in triplicate and reported as mean ± standard deviation. *P* < 0.05 (*), *P* < 0.01 (**), *P* < 0.001 (***), *P* < 0.0001 (****).

Calcification was also studied after 28 days of incubation in the same experimental setups to check how the experimental groups have affected the mineralization of extracellular matrix in the long term. First, the samples were stained with an alizarin red solution. This kit is based on the alizarin red molecule making a complex with Ca ions in the acidic pH range. In the nonstimulated and co-conditioned (immune-stimulated) models, none of the experimental groups were able to stimulate calcification compared to the control (Figure 6a, b). There was also no significant difference between icariin loaded and nonloaded groups. This observation can be explained based on the low values of ALP in these groups. In the co-culture model, indirect incubation of the BG5% loaded with icariin (BG5%-Ic) group could increase the trapped alizarin red molecules (Figure 6c). The remaining nanoparticles in the released supernatant, which could be phagocytized by THP-1 derived macrophages, can explain this observation. Comparing the co-conditioned and co-culture models, it is worth highlighting the advantages and disadvantages of the models. Macrophage population, macrophage viability, and the cross-talk between hMSCs and macrophages are the important factors to consider. As previously mentioned, macrophages get initially recruited to the site of bone defect due to the immune response to foreign bodies. The population of macrophages decreases over time, leading to the regeneration phase.^34^ The co-cultured model lacks this variation in the population of macrophages. On the other hand, limited viability of macrophages can affect the immune response due to dead bodies. However, the co-culture model presents the feedback loop between hMSCs and macrophages. Earlier studies have proved the anti-inflammatory effect of MSCs on macrophages, which plays a role in the regeneration process.^35^

Alizarin red is not specific to hydroxyapatite and forms a complex with Ca and Cu ions in the copper-doped BGNPs.^36^ The colloidal stability is the main parameter that can affect the suspended nanoparticles after centrifugation. On the other hand, coating nanoparticles with branched polymers will increase the colloidal stability and reduce the hydrodynamic diameter, known as steric stability.^37^ To investigate the hydrodynamic diameter of the nanoparticles, DLS was applied to loaded and nonloaded samples in PBS (Table 3). Similar particle size and surface charge (Figure 3d) in BG, BG3% and BG5% samples led to nonsignificantly different hydrodynamic diameters. On the other hand, nHA showed a smaller hydrodynamic diameter due to the smaller particle sizes. After icariin loading, the hydrodynamic diameter increased in the BG-Ic samples. This is because of the change in surface charge from negative to neutral that leads to the elimination of electrostatic stability. In the BGNPs doped with copper, an increase in Cu content ended in a decreased hydrodynamic diameter in icariin-loaded samples. Chelate formation of icariin with Cu ions on the surface can increase the steric stability, leading to a decrease in the hydrodynamic diameter. Therefore, the low diameter of BG5%-Ic aggregates can explain the residue particles in the released supernatant, increased uptake of nanoparticles by THP-1 derived macrophages and consequently artificial increase in alizarin red chelates in the well plate.^38^

**Table 3:** Hydrodynamic Diameter of the Nanoparticles without and with Icariin Loading

Sample	Hydrodynamic diameter without icariin (nm)	Hydrodynamic diameter with icariin (nm)
**BG**	192 ± 24	332 ± 45
**BG3%**	217 ± 28	143 ± 18
**BG5%**	258 ± 32	70 ± 9
**nHA**	105 ± 7	107 ± 7

**Figure 7:**
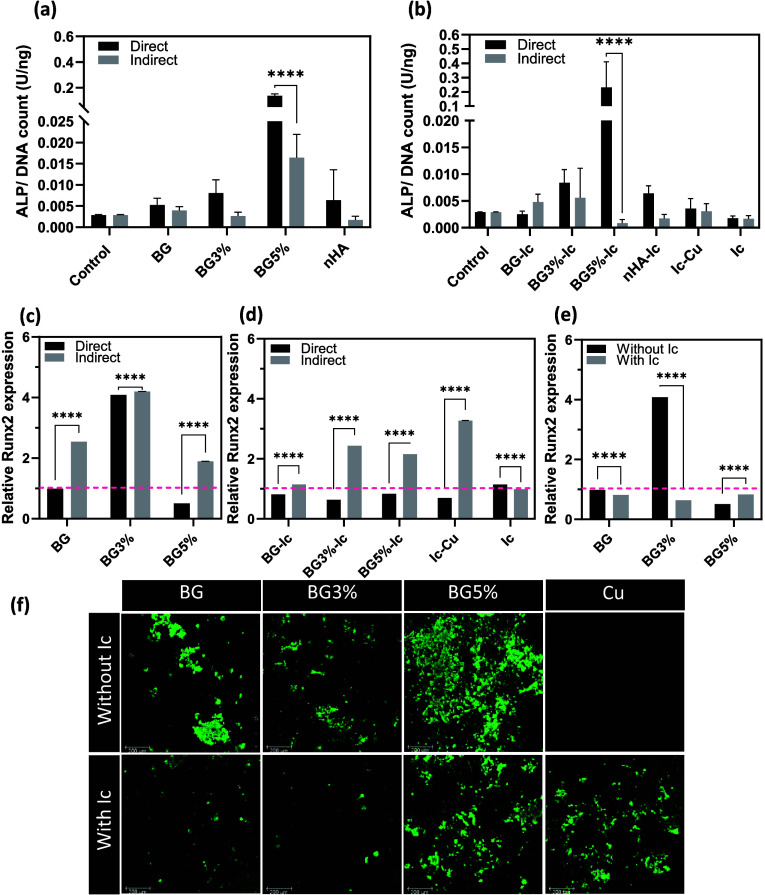
ALP normalized to picogreen (DNA count) of hMSCs incubated with (a) nonloaded and (b) icariin-loaded nanoparticles in the co-culture in vitro model. Runx2 gene expression of hMSCs incubated directly and indirectly with (c) nonloaded and (d) icariin-loaded nanoparticles in the co-culture in vitro model. (e) Runx2 expression of cells incubated directly with nonloaded and icariin-loaded nanoparticles in the co-culture vitro model. These values were normalized to the control (pink dash line). (f) Osteoimages of the co-culture model directly incubated by the experimental groups: calcified hydroxyapatite stained in green (scale bar is equal to 200 μm). All measurements for parts (a) to (e) were done in triplicate and reported as mean ± standard deviation. *P* < 0.05 (*), *P* < 0.01 (**), *P* < 0.001 (***), *P* < 0.0001 (****).

Regarding direct co-cultured samples (Figure 6d), the signal intensity of CPC dissolved alizarin red was increased by 1 order of magnitude. Compared to indirect co-cultured samples, BG5%-Ic, BG5%, HA, and HA-Ic showed the highest signal. This result can be partially attributed to cellular uptake in BG5%-Ic, HA and HA-Ic samples by having smaller hydrodynamic diameter. The osteoimage kit as a hydroxyapatite-specific kit was used to exclude the mentioned artificial effect. The reactive fluorescent chemical in this kit interacts with phosphate units instead of Ca, so inorganic substitutes such as BGNPs and also icariin do not affect the results based on the manufacturer. The results of the direct experiment in the co-culture model showed that icariin-loaded BG and nHA promoted calcification to a higher extent than bare samples. However, the results are not reliable for nHA due to artificial staining of the nanoparticles from the sample. Although Ic-Cu showed osteogenic properties, the Cu-doped BGNPs worked better without the presence of icariin (Figure 6e). This shows that the free Cu ions have a higher osteo-immunomodulatory effect compared to Ic-Cu. In the groups exposed to osteogenic medium, ALP expression, Runx2 gene expression and calcification were studied after 14 days of culture. ALP activation was shown to be higher once the nanoparticles were directly exposed to the co-culture model. Among all the icariin loaded and nonloaded experimental groups, BG5% and BG5%-Ic showed the highest ALP activation (Figure 7a, b). However, Runx2 expression followed a different pattern by showing higher gene expression in indirectly incubated samples (Figure 7c, d). Among all experimental groups, BG3% showed the highest Runx2 expression. Loading icariin in BG3%, however, decreased the gene expression dramatically (Figure 7e). Calcification in the samples followed the ALP results and was shown to be the highest in the co-culture model directly incubated with BG5% (Figure 7f).

**Figure 8:**
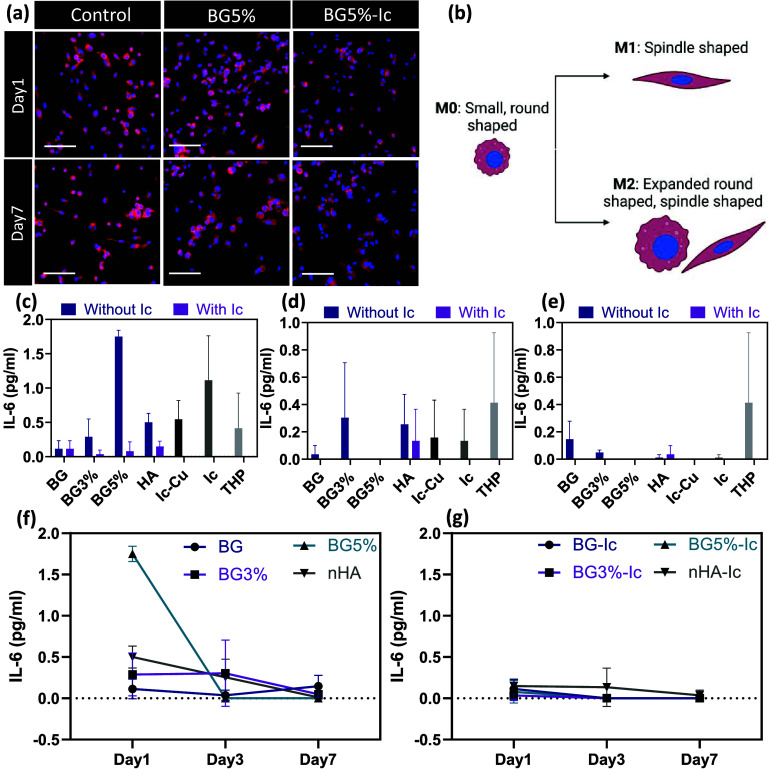
(a) DAPI- phalloidin stained THP-1 derived macrophages stimulated by released supernatants of BG5% and BG5%-Ic, captured by confocal microscopy (scale bar is equal to 100 μm). (b) Morphologies of M0, M1 and M2 phenotypes of THP-1 derived macrophages. The concentration of secreted IL-6 cytokine after incubation with released supernatant after (c) 1, (d) 3, and (e) 7 days of incubation. The change in IL-6 levels in (f) nonloaded and (g) icariin-loaded nanoparticles over time. All the measurements for parts (c) to (g) were done in triplicate and reported as mean ± standard deviation. *P* < 0.05 (*), *P* < 0.01 (**), *P* < 0.001 (***), *P* < 0.0001 (****).

Immune response to the studied experimental groups can clear the role of icariin and Cu and the chelation of both. First, THP-1 derived macrophages were exposed to BG5% and BG5%-Ic and the morphology of the cells was studied after cytoskeleton staining and confocal imaging. It was previously established that different phenotypes of THP-1 derived macrophages have different morphologies. The M0 phenotype has a small round shape; however, its morphology transfers to a spindle shape with increased pseudopodia once it changes to the M1 phenotype (pro-inflammatory), whereas the M2 (anti-inflammatory) phenotype shows larger expanded macrophages that are spindle or round shaped.^39^ As is visible in Figure 8a, b, the nontreated THP-1 macrophages had a combination of small round and spindle-shaped cells. This shows that the cells were still affected by the passaging and differentiating procedures, turning some cells into the M1 phenotype. Adding the released supernatant of BG5% after 24 h of incubation showed an increase in M1 phenotype (spindle-shaped) while the BG5%-Ic did not affect the morphology considerably. The release of supernatant of the nanoparticles after 7 days returned the morphology into an expanded round shape (M2 phenotype) again. This transition was more significant in BG5%-Ic. This result shows that the release of Cu ion causes inflammation after 24 h; however, icariin as an anti-inflammatory drug not only has suppressed the inflammation but also pushed cells into an anti-inflammatory phenotype. To quantify the intensity and duration of the inflammation phase, IL-6 cytokine secretion was quantified using an ELISA kit. Although BG5% caused the highest concentration of IL-6 during the first day, BG5%-Ic suppressed IL-6 compared to the control (THP) due to releasing icariin. Free icariin also caused increased IL-6 secretion because of the high and toxic concentration of the drug in this sample (Figure 8c). During 7 days, the IL-6 concentration decreased in all samples (Figure 8f, g).

It is necessary to look into the role of inflammation in bone regeneration to be able to explain the correlation between the calcification and immunogenicity results. The bone regeneration procedure can be divided into three phases, namely inflammation, repair and remodeling.^40^ The inflammation phase, which involves neutrophils and macrophages at the site of the defect, lasts a few days. Pro-inflammatory cytokines and chemokines recruit MSCs to the site of the defect and induce osteogenesis via the COX-2-PGE2 pathway.^41,42^ The beneficial role of initial inflammation in bone regeneration has been also shown earlier in vivo.^43^ On the other hand, chronic inflammation has been proven to have a destructive effect by stimulating osteoclastogenesis.^44^ Having these facts in mind, the immunomodulatory effect of Cu ion and icariin becomes clearer. It is also important that Cu and icariin have direct intrinsic osteogenic properties which can be affected/covered by their osteo-immunogenic properties.^45,46^ As mentioned, Cu ions showed the capability to raise the pro-inflammatory microenvironment in the short term. This characteristic helps to mimic the first phase of bone regeneration. Loading BG5% with icariin decreased inflammation just after including nanoparticles. The negative consequence of this phenomenon was proven by a calcification study. Strong bonding between icariin and Cu ions might be a reason to explain the suppression of the pro-inflammatory effect of Cu ions.

The primary unresolved question may be why Runx2 expression is reduced in samples with high ALP activity and extensive calcification. Although Runx2 fluctuations due to secreted inflammatory cytokine showed dose dependent results in multiple studies, it was shown by Ding et al. that TNF-α (0.1 to 10 ng/mL) and IL-1β (0.1 to 1 ng/mL) can decrease Runx2 levels and in contrast increase ALP and calcification in osteogenic differentiated MSCs.^47^ This can be explained by the fact that TNF-α promotes Runx2 degradation.^48^ Therefore, ALP activity and mineralization do not always follow the Runx2 activity trend. Similar to TNF-α, IL-6 also showed similar effects based on the current reported data.

The immunomodulations as studied in the current in vitro models (co-conditioned and co-cultured) have drawbacks which can be tackled. The studies showed that hMSCs have immunomodulatory capability themselves, an effect that cannot be recognized in the co-conditioned model.^49^ In the case of the co-culture model, the fluctuations in the population of macrophages during different phases of bone regeneration cannot be appropriately mimicked.^50^ As the present study has shown, due to the high number of variables, designing the experiment and adjusting the time points is highly critical to collect valid results.^51^

## 4 Conclusions

This study has investigated the osteogenic and osteo-immunogenic properties of copper ion and icariin as two agents that can potentially be used in bone regeneration. Although the osteogenic properties and immunogenic properties of these agents have been studied before, their joint effect has been left unexplored. In this regard, mesoporous bioactive glass nanoparticles doped with copper ions were designed and synthesized as the bioactive carrier for both Cu ions and icariin. Three in vitro models were developed to mimic in vivo conditions as closely as possible. An early marker of ALP showed that the released copper ions increased osteo-immunogenesis, while icariin, as an anti-inflammatory drug, reversed this effect. Although calcification studies confirmed this fact for copper-loaded nanoparticles, the results were again reversed for samples without copper. Knowing Cu and icariin are intrinsic osteogenic agents, osteogenesis and osteo-immunogenesis might compete or cooperate. It was shown that copper has a stronger osteo-immunogenic effect compared with the direct osteogenic effect of icariin. However, icariin increased ALP activation around two times more than released Cu ions without immune stimulation.

Altogether, BGNPs doped with 5% Cu caused the highest level of bone formation in the presence of macrophages. The immune response of macrophage-like THP-1 cells proved the pro-inflammatory effect of copper ions and the anti-inflammatory effect of icariin. These results confirmed that mimicking the inflammation fluctuations in the bone healing timeline stimulates bone regeneration. This study showed that adding icariin into Cu-containing samples reduces osteo-immunogenesis due to the anti-inflammatory effect of icariin and reduction of free Cu concentration by forming the icariin-Cu chelate. This study indicated the importance of multicellular in vitro models to decrease the gap between in vitro and in vivo outcomes. Innate immune cells seem to be the key components of these in vitro scenarios. Overall, these multicellular in vitro models with hMSCs and innate immune cells elucidate the osteo-immunogenic effects of the adjuvants Cu and icariin loaded in bioactive glass nanoparticles. Both adjuvants alone improved the osteo-immunogenic responses. However, in combination, icariin diminished the positive effects of Cu. Bioactive glass nanoparticles with Cu alone provided the best osteogenic results.
